# Downhill running regulates cardiac immune response through GCN2

**DOI:** 10.1371/journal.pone.0329973

**Published:** 2025-08-22

**Authors:** Xinyue Bai, Wanting Zhang, Yingyue Zhang, Xin Xu

**Affiliations:** 1 School of Exercise and Health, Shanghai University of Sport, Shanghai, China; 2 Research Institute for Doping Control, Shanghai University of Sport, Shanghai, China; Livingstone Center for Prevention and Translational Science, ZAMBIA

## Abstract

Downhill running (DR) has recently emerged as a promising exercise modality for cardiac rehabilitation, but the effect and mechanism of DR on myocardium remains unclear. General control nonderepressible 2 (GCN2), an eukaryotic initiation factor 2α (eIF2α) kinase, is beneficial to the heart when it is deficiency. The current study aimed to explore whether the GCN2 is associated with cardioprotective effects in downhill running. Eight-week-old male wild type (WT) mice and GCN2 knock out (KO) mice were randomly divided into WT control (n = 6), WT exercise(n = 5), GCN2 KO control(n = 5), and GCN2 KO exercise(n = 5) groups. Mice in the exercise groups were subjected to a single session of downhill running treadmill exercise training. In WT mice, DR increased the proportion of basophils, decreased the percentage of lymphocytes in the blood, and decreased the expression of GCN2, *Ifn-γ*, **Tgf-*β*r1** and immune cell markers in the myocardium. Compared with WT mice, GCN2 KO mice decreased the proportion of monocytes and neutrophils in the blood, decreased expression of *Ifn-γ*, **Tgf-*β*r1** and immune cell markers, and increased expression of *Il-12α* in the myocardium. In GCN2 KO mice, DR increased the expression of immune cell markers in the myocardium. DR and GCN2 KO both reduced TGF-β1 expression, and elevated p-eif2a expression in the myocardium. This finding demonstrated that downhill running alters inflammation and immune response in the myocardium, which is associated with GCN2.

## Introduction

Cardiovascular disease has become a major health problem with a huge economic and health burden globally [1]. Cardiac rehabilitation is a comprehensive intervention based on a holistic medical assessment, with the key goals including improving the patient’s exercise capacity and health-related quality of life, as well as adjunctive medications to reduce the risk of hospitalization and death [2,3]. Downhill running (DR), a type of eccentric exercise, has gained increasing attention as a suitable and promising intervention for positive rehabilitation and training effects [4,5]. The characteristic of eccentric exercise is the ability to produce greater mechanical loads, cause little cardiovascular stress with lower cardiorespiratory demands than concentric exercise [6,7]. Eccentric exercise is effective in improving cardiovascular fitness and related markers compared to traditional exercise modalities [8]. Eccentric and endurance exercise induced less myocardial apoptosis compared to suspension and resistance exercise [9]. There are many studies out there on the link between DR and muscle inflammation [10,11]. Less is known, however, regarding the effects of DR on the myocardium.

General control nonderepressible 2 (GCN2) is a serine/threonine protein kinase, which is one of the stress kinases that block translation by phosphorylating eIF2α [12]. In general, GCN2 activity in cells must be efficiently inhibited to allow maximum rates of protein synthesis [13]. GCN2 deficiency protects cardiac function in cardiac dysfunction mice by reducing lipid accumulation, myocardial fibrosis, oxidative stress, and cell death [14,15]. Also, exercise preconditioning has been reported to protect against acute cardiac injury via GCN2 [16]. Emerging evidence reveals that GCN2 is closely linked to the immune system and is essential in the maintenance of immune homeostasis [12], such as GCN2 plays an important role in regulate control macrophage functional polarization and CD4^+^ T cell subset differentiation [13]. However, to date, GCN2-induced myocardial immune responses have not been evaluated.

Therefore, considering the close relationship between immune cells and GCN2, and the potential advantages of DR in attenuating the load of heart, we evaluated the effects of DR on inflammatory response, immune response, and GCN2/eIF2α pathway before and after knockout of the GCN2 gene, respectively.

## Materials and methods

### Animals

All animal experiments were approved by Shanghai University of Sport Animal Care and Use Committee and were in accordance with the National Institutes of Health guidelines (Ethics No. 102772024DW022). Eight-week-old male WT mice (n = 11) were purchased from Shanghai JieSiJie Laboratory Animal Co.,Ltd. (Shanghai, China). Eight-week-old male GCN2 KO mice (n = 10) were purchased from the Jackson Laboratory (Bar Harbor, ME, USA). Mice were housed in a 12-h light/12-h dark animal room with free access to sterilized water and food. Animal room was maintained under standard conditions of temperature 20–24°C and 45–55% humidity. All the animals were randomly divided into four groups: WT control group (C), WT exercise group (E), GCN2 KO control group (G), GCN2 KO exercise group (GE).

The animals in the exercise groups were subjected to a single session of DR on a motor treadmill (−16° slope) at moderate intensity (16 m/min) for 90 min. The heart of mice in each group were collected 48 h right after exercise. All mice were injected intraperitoneally with 1.25% tribromoethanol anesthetic, and all efforts were made to minimize suffering.

### Detection of inflammatory cell levels

To assess the effects of exercise and GCN2 on the populations of Inflammatory cells in blood, routine blood test was performed for both WT and GCN2 KO mice. All mice were euthanized by Tribromoethanol and sacrificed for the further study. Blood was collected by abdominal aortic puncture in EDTA blood collection tubes from mice at the day of sacrifice. The blood cells of the mice were analyzed by using animal automatic hematology analyzer.

### Quantitative real-time polymerase chain reaction (*RT-qPCR*)

Total RNA was extracted from each myocardium sample using the FastPureR Cell/Tissue Total RNA Isolation Kit V2 (Vazyme, Lot # RC112–01). The purity and concentration of the extracted RNA were determined by nano drop (Thermo Fisher Scientific, Lot # 701–058112). Reverse transcriptional reactions were performed using the RevertAid First Strand cDNA Synthesis Kit (Thermo Fisher Scientific, Catalog # K1622). The cDNAs were amplified with SYBR green master mix (Vazyme, Lot # Q712-02), with *Gapdh* as an endogenous control.

RT-PCR was performed on QuantStudio 6 Flex (Thermo Fisher Scientific) using with the following parameters: initial denaturation at 95°C for 30s followed by 40 cycles of 10s at 95°C, and 30s at 60°C for annealing and 15s at 95°C, 60s at 60°C, and 15s at 95°C for extension. The RT-PCR was done in triplicate and repeated at least 3 times. Primers are listed in Table 1.

**Table 1:** Primers sequences (5’- 3’).

Gene	Forward	Reverse
*Gapdh*	AGGTCGGTGTGAACGGATTTG	TGTAGACCATGTAGTTGAGGTCA
*Il-10*	CTTACTGACTGGCATGAGGATCA	GCAGCTCTAGGAGCATGTGG
*Tgf-βr1*	TCCCAACTACAGGACCTTTTTCA	GCAGTGGTAAACCTGATCCAGA
*Tgf-βr2*	TTGGATTGCCAGTGCTAACCC	AACAAGCCACAGTAACATGACA
*Il-6*	TAGTCCTTCCTACCCCAATTTCC	TTGGTCCTTAGCCACTCCTTC
*Il-1β*	GAAATGCCACCTTTTGACAGTG	TGGATGCTCTCATCAGGACAG
*Il-12α*	CTGTGCCTTGGTAGCATCTATG	GCAGAGTCTCGCCATTATGATTC
*Tnf-α*	CCCTCACACTCAGATCATCTTCT	GCTACGACGTGGGCTACAG
*Nf-κb*	ATGGCAGACGATGATCCCTAC	TGTTGACAGTGGTATTTCTGGTG
*Ifn-γ*	ATGAACGCTACACACTGCATC	CCATCCTTTTGCCAGTTCCTC
*Cd3*	ATGCGGTGGAACACTTTCTGG	GCACGTCAACTCTACACTGGT
*Cd8*	CCGTTGACCCGCTTTCTGT	CGGCGTCCATTTTCTTTGGAA
*Cd69*	CCCTTGGGCTGTGTTAATAGTG	AACTTCTCGTACAAGCCTGGG
*Lag3*	CTGGGACTGCTTTGGGAAG	GGTTGATGTTGCCAGATAACCC
*Nkg2d*	ACTCAGAGATGAGCAAATGCC	CAGGTTGACTGGTAGTTAGTGC
*Nkp46*	ATGCTGCCAACACTCACTG	GATGTTCACCGAGTTTCCATTTG
*Cd56*	GTACTCGGTACGACTGGCG	TGGAGGAGGGCTATGGACTG
*Inos*	GTTCTCAGCCCAACAATACAAGA	GTGGACGGGTCGATGTCAC
*Cd68*	TGTCTGATCTTGCTAGGACCG	GAGAGTAACGGCCTTTTTGTGA
*Cd206*	CTCTGTTCAGCTATTGGACGC	CGGAATTTCTGGGATTCAGCTTC
*Cd209*	CTGGCGTAGATCGACTGTGC	AGACTCCTTGCTCATGTCAATG
*Cd15*	ACGGATAAGGCGCTGGTACTA	GGAAGCCATAGGGCACGAA

*Gapdh*, Glyceraldehyde 3-phosphate dehydrogenase; *Il-10*, Interleukin 10; **Tgf-*β*r1**, transforming growth factor-β receptor 1; **Tgf-*β*r2**, transforming growth factor-β receptor 2; *Il-6*, interleukin 6; *Il-1β*, interleukin 1 beta; *Il-12α*, interleukin 12 alpha; *Tnf-α*, tumor necrosis factor–alpha; **Nf-*κ*b**, nuclear factor kappa-light-chain-enhancer of activated B cells; *Ifn-γ*, interferon gamma; *Cd3*, cluster of differentiation 3; *Cd8*, cluster of differentiation 8; *Cd69*, cluster of differentiation 69; *Lag3*, lymphocyte activation gene-3; *Nkg2d*, natural killer group 2 member D; *Nkp46*, natural cytotoxicity receptor 46; *Cd56*, cluster of differentiation 56; *Inos*, inducible nitric oxide synthase; *Cd68*, cluster of differentiation 68; *Cd206*, cluster of differentiation 206; *Cd209*, cluster of differentiation 209; *Cd15*, cluster of differentiation 15.

### Western blot

Total protein was extracted from heart tissue with RIPA buffer (Beyotime, P0013B), protease and phosphatase inhibitor cocktail (Beyotime, P1045). The total protein concentration was measured using a BCA protein assay kit (Beyotime, P0012). The extracted proteins were separated via sodium dodecyl sulfate–polyacrylamide gel electrophoresis (SDS-PAGE) and transferred onto polyvinylidene difluoride (PVDF) membranes (Millipore, Billerica, MA). The membrane was blocked in Protein Free Rapid Blocking Buffer (Vazyme, PS108P) for 15 min at room temperature. Then incubated at 4 ◦C overnight with primary antibodies, including anti-IL-6 (1:1000, abcam), anti-TNF-α(1:1000, CST), anti-IL-10 (1:1000, abcam), anti-TGF beta 1 (1:1000, abcam), anti-GCN2 (1:1000, CST), anti-eif2α(1:1000, CST), anti-p-eif2α(1:1000, CST) and anti-GAPDH (1:50000, proteintech). The membranes were washed with TBST and incubated with HRP-conjugated goat anti-mouse (Proteintech, SA00001−1) or anti-rabbit (ZSGB-BIO, SA00001−1) secondary antibodies for 1.5 h the next day. Then the membranes were washed and exposed using an imaging system (BIO-RAD, Tanon-5200S). ECL kit (Biosharp, BL520A) was used to produce a signal to visualize the protein bands. The captured images were analyzed using Image-J software, and the values of target proteins were normalized to that of the internal control protein on the same membrane.

### Statistics and data analysis

Data were analyzed with GraphPad Prism 8.0. Results are presented as mean ± SD. All figures were made using GraphPad Prism 8.0. All data were tested for normality and homogeneity before statistical analysis using Shapiro-Wilk test. Statistical comparisons were done with a Two-way analysis of variance plus Tukey post hoc tests, and the Kruskal-Wallis test with Dunn correction. The level of significance was set at **p* *< 0.05.

## Results

### DR and GCN2 KO affect inflammatory cells and cytokines

**Fig 1:**
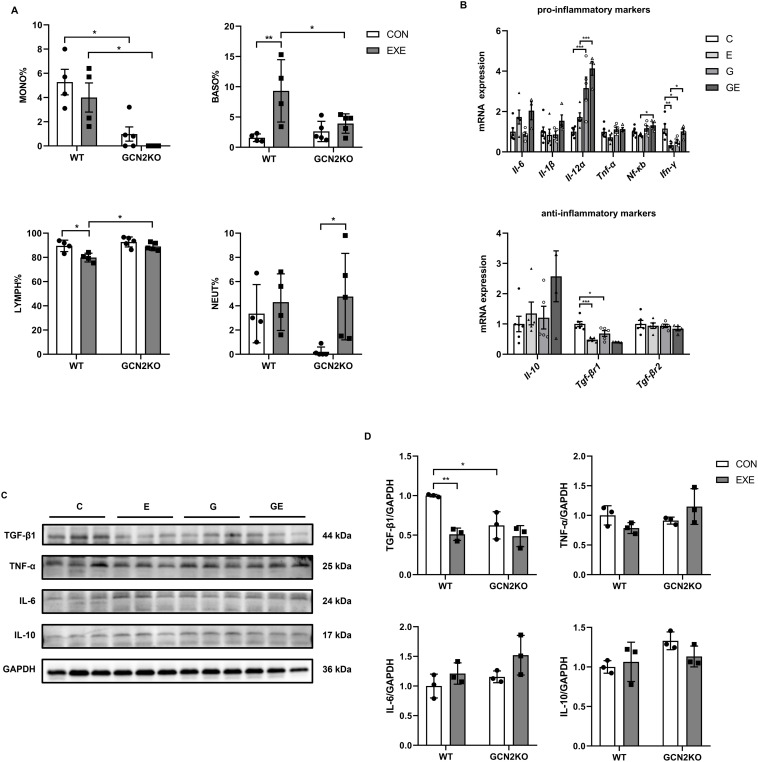
Effects of downhill running and GCN2 KO on inflammation levels. **(A)** The blood profile of mice after downhill running or GCN2 KO. The percentage of Monocyte, Basophilic granulocytes, Lymphocyte and Neutrophils including in blood (n = 4-5). **(B)** The mRNA expression level of pro-inflammatory cytokines, *Il-6*, *Il-1β*, *Il-12α*, *Tnf-α*, **Nf-*κ*b**, *Ifn-γ*, and of anti-inflammatory cytokines, *Il-10*, **Tgf-*β*r1**, and **Tgf-*β*r2** (n = 4-6). **(C)** The protein expression of TGF-β1, TNF-α, IL-6, and IL-10 in myocardium were detected by western blotting. **(D)** Comparison of protein expression in each group (n = 3). Data are expressed as means ± SD, *p < 0.05, **p < 0.01, ***p < 0.001, Cohen’s d > 0.8.

The routine blood test found that in WT mice, DR increased the percentage of basophilic granulocytes and decreased the percentage of lymphocytes (Fig 1A). Compared with WT mice, GCN2 KO mice showed a significant decrease in the proportion of monocytes and the same trend in the proportion of neutrophils (Fig 1A).

In order to investigate the expression profile associated with an inflammatory response, we examined the expression levels of inflammation-related factors in myocardial tissues. It was found that in WT mice, acute DR induced significant decrease in *Ifn-γ* and **Tgf-*β*r1** (Fig 1B). We next determined whether GCN2 would influence inflammation cytokines in myocardium. GCN2 KO attenuated the expression of *Ifn-γ* and **Tgf-*β*r1** (Fig 1B) compared with WT group. By contrast, the expression of pro-inflammatory cytokine *Il-12α* was obviously increased by knockout of GCN2 (Fig 1B). Moreover, compared with WT exercise mice, the expression of *Il-12α*, **Nf-*κ*b**, *Ifn-γ* were significantly increased in myocardium of GCN2 KO mice under the treatment of DR (Fig 1B).Consistent with the trend in gene expression, western blot analysis showed DR and GCN2 KO both decreased the expression of TGF-β1 and there were no differences in IL-10, IL-6, or TNF-α protein expression regardless of downhill running or GCN2 KO (Fig 1C and 1D).

### WT mice Presents a Decrease of the Immune Response After DR, while GCN2 KO mice Presents an Increase

**Fig 2:**
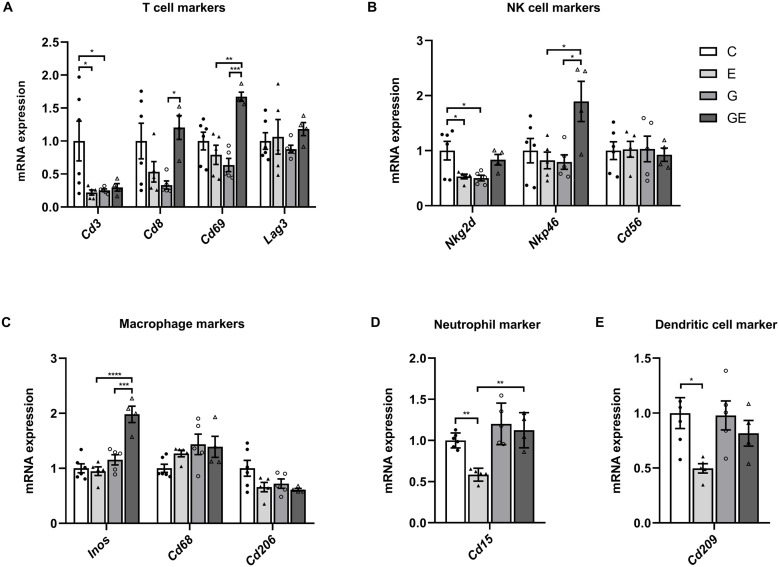
The effects of downhill running and GCN2 KO in the myocardial of immune cell markers. The mRNA levels of **(A)** T cell markers, **(B)** NK cell markers, **(C)**Macrophage markers, **(D)**Neutrophil marker and **(E)** Dendritic cell marker were determined in mice myocardium (n = 4-6). Data are expressed as means ± SD, *p < 0.05, **p < 0.01, ***p < 0.001, ****p < 0.0001, Cohen’s d > 0.8.

It was found that DR significantly decreased the expression of *Cd3*, *Nkg2d*, *Cd15* and *Cd209* in WT mice (Fig 2A, 2B, 2D, and 2E). The expression of *Cd3* and *Nkg2d* in GCN2 KO mice was lower than in WT mice (Fig 2A and 2B). In GCN2 KO mice, transcript levels of immune cell markers such as T cells (*Cd8*, *Cd69*), NK cells (*Nkp46*) and macrophages (*Inos*) were significantly elevated in DR mice (Fig 2A-2C). It is suggested that T cell, NK cell, and macrophage in the myocardium are more sensitive to exercise and can be activated, trafficking more after GCN2 KO. In addition, compared with WT exercise mice, immune cell markers were significantly increased in myocardium of GCN2 KO mice under the treatment of DR. The results showed that the T cells, NK cells, pro-inflammatory M1-like macrophages, and neutrophil were elevated, as demonstrated by increased levels of the markers of immune cells, such as *Cd69*, *Nkp46*, *Inos* and *Cd15* (Fig 2A-2D).

### DR attenuates myocardial GCN2 expression and elevates p-eIF2α expression

**Fig 3:**
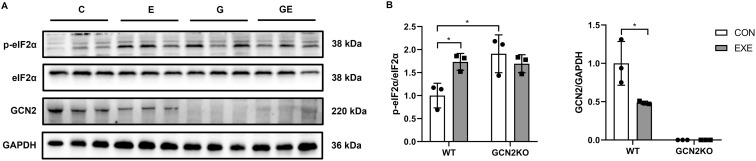
Effects of downhill running on GCN2/eIF2α pathways in WT and GCN2 KO mice. **(A)** Western blot detected the expression of p-eIf2α, eIF2α and GCN2 with GAPDH as an internal reference. **(B)** Comparison of protein expression in each group (n = 3). Data are expressed as means ± SD, *p < 0.05, **p < 0.01, Cohen’s d > 0.8.

To determine whether the GCN2 affects DR induced immune response through eIF2α pathway, we evaluated the levels of GCN2, p-eIF2α, and eIF2α in the myocardium of mice at 48h after DR. It was found that the expression of GCN2 protein was lower in DR mice than in WT mice (Fig 3), suggesting that exercise preconditioning decreased myocardial GCN2 expression. Although GCN2 is an eIF2α kinase, downhill running significantly elevated myocardial eIF2α phosphorylation under basal conditions (Fig 3). Moreover, GCN2 KO also increases p-eIF2α protein expression (Fig 3).

## Discussion

The main finding of this study is that acute downhill running alters immune cells and cytokines in the myocardium, which is associated with GCN2/eIF2α pathway.

The body responds to exercise via complex adaptive processes to maintain cardiac function. Central to this process is inflammation and immune cell signaling [17]. Immune system provides the protective inflammatory response that the host needs to fight off infection [18]. A growing body of research suggests that interleukins, neutrophils, and inflammasome are associated with the inflammatory process of cardiac injury [19]. It has been reported that serum neutrophils were elevated 4 hours after DR [20], and muscle neutrophils were elevated 24 hours after DR [21]. Emerging evidence suggested that acute graded exercise stress test significantly increased lymphocyte, T cell, and CD8^+^ T cell counts in peripheral blood, while 6 weeks of eccentric exercise was not sufficient to affect changes in lymphocyte and T-cell subsets [22]. The results of the study suggested an increase in the proportion of basophils and a decrease in the percentage of lymphocytes in the blood after 48 hours of DR, with no change in monocytes or neutrophils. It is suggested that blood is redistributed after exercise, and the body’s immune cells may migrate through the bloodstream to different tissues at different points in time.

It has been well accepted that DR is an effective method for rehabilitation and training, as it causes little cardiovascular stress with lower cardiorespiratory demands [6,7]. However, there are fewer studies on the effects of DR on the heart. Our findings revealed that *Ifn-γ*, **Tgf-*β*r1** and immune cell (T cell, NK cell, dendritic cell and neutrophil) markers in the myocardium decreased after a single bout of DR. It suggested that cytokines can interact with immune cells to regulate cardiac adaptation to various physiological stresses.

GCN2, one of four stress kinases in the body that regulate the amino acid starvation response, is closely linked to the immune system and is essential in the maintenance of immune homeostasis [12]. Our results revealed that GCN2 KO decreased the proportion of monocytes in the blood, and the same trend was observed for the percentage of neutrophils. Normally, the immune system senses pathogens through pathogen recognition receptors, but emerging evidence suggests that it can also sense and response to cellular stresses [23]. When GCN2 is deficient, it affects the release of inflammatory cytokines [24]. Consistent with the effects of downhill running, results in the myocardium showed that GCN2 deficiency decreases the expression of *Ifn-γ*, **Tgf-*β*r1**, and immune cell markers, whereas *Il-12α* expression is elevated. In addition, cardiac pressure overload activates the TGFβ cascade, which can initially be protective. However, chronically overactive TGFβ signaling in pressure-overloaded cardiomyocytes promotes cardiac fibrosis and dysfunction [25]. Thus, both DR and GCN2 KO reduce TGF-β1 expression may be cardioprotective.

Immune cells play important roles in cardiac homeostasis and response to stress [17]. T cells, NK cells, and macrophages have been shown to benefit cardiac repair [17,26]. It has been reported that GCN2 is required for the proliferation and trafficking of cytotoxic T cells [27], which is opposite of what we observed. In the case of GCN2 deficiency, we observed that downhill running caused the up-regulation of T cells, NK cells, and M1 macrophages expression in the myocardium. This suggests that after GCN2 KO, DR induces the release of cell signaling molecules from the myocardium, and mobilizes more immune cells to exert cardioprotective effects. Dendritic cells (DCs) are antigen-presenting cells that orchestrate innate and adaptive immune responses [28]. DCs sense pathogens and signals through pattern recognition receptors, deliver antigens to helper T cells receiving instructions, helper T cells activate cytotoxic T cells, natural killer cells, macrophages and release inflammatory cytokines to initiate innate immune responses [23,28]. The results suggested that GCN2 KO may attenuated the inhibition of DC, and DR after GCN2 KO may activating the expression of T cells, NK cells and M1 macrophages in the myocardium, thus exerting an immune effect to protect the myocardium.

Sun et al. reported that exercise preconditioning led to a decrease in cardiac GCN2 expression, which was beneficial in improving cardiac dysfunction and inflammation and reducing cardiac injury [16]. In agreement with this, in the current study, the expression of myocardial GCN2 decreased after DR. As eIF2α is the only identified substrate for GCN2, GCN2 deficiency attenuates cardiotoxicity by reducing apoptosis and oxidative stress via eIF-2α/CHOP-dependent pathway [14], we observed p-eif2a expression in myocardium. In fact, we found that DR and GCN2 KO both elevate p-eif2a expression. The eIF2α upstream kinases PERK, PKR, GCN2, and HRI modulate protein synthesis and trigger integrated stress responses (ISR) through phosphorylation eIF2α [29,30]. In the acute phase of ISR, phosphorylation of eIF2α reducing unfolded protein stress, which is beneficial for maintaining cellular function [31]. Though DR reduces myocardial GCN2 expression, other kinases may exert a protective effect by compensatory activation driving eif2a phosphorylation and adapting cardiomyocytes to stress. Therefore, more studies are needed to address this point.

There are some limitations in our work. First, flow cytometry was not performed in this study, which allows for more direct detection of changes in immune cell markers. In addition, the benefit of GCN2 KO in modulating cardiac immune function after DR was not assessed at the protein level and future studies are needed to evaluate these questions. Moreover, repetitive DR training has been reported to have a sustained protective effect on the muscle [32,33], which make a hint that the relationship between repetitive DR and the myocardium is worth exploring. Therefore, the benefits of DR on cardiac immune function have not been fully evaluated and more investigations are needed to elucidate the comprehensive mechanisms.

In conclusion, the present investigation confirmed that a single bout of DR can affect myocardial immune cells and cytokines by a mechanism that may be related to the GCN2/eif2α pathway. Importantly, knockout of GCN2 increases the recruitment of myocardial immune cells after exercise. In addition, DR and GCN2 KO both alleviated the TGF-β1 level and elevated p-eif2a expression, which may be cardioprotective.

## Supporting information

S1 Fig S1_raw_image. Original images for blot and gel.
(PDF)

S1 File S1_data. All raw datasets.
(XLSX)
