# Supplementary figures and images for: Downhill running regulates cardiac immune response through GCN2
Source: PLoS One. 2025 Aug 22;20(8):e0329973. doi: 10.1371/journal.pone.0329973 (PMC12373192; doi:10.1371/journal.pone.0329973)

Original image for Fig 1C

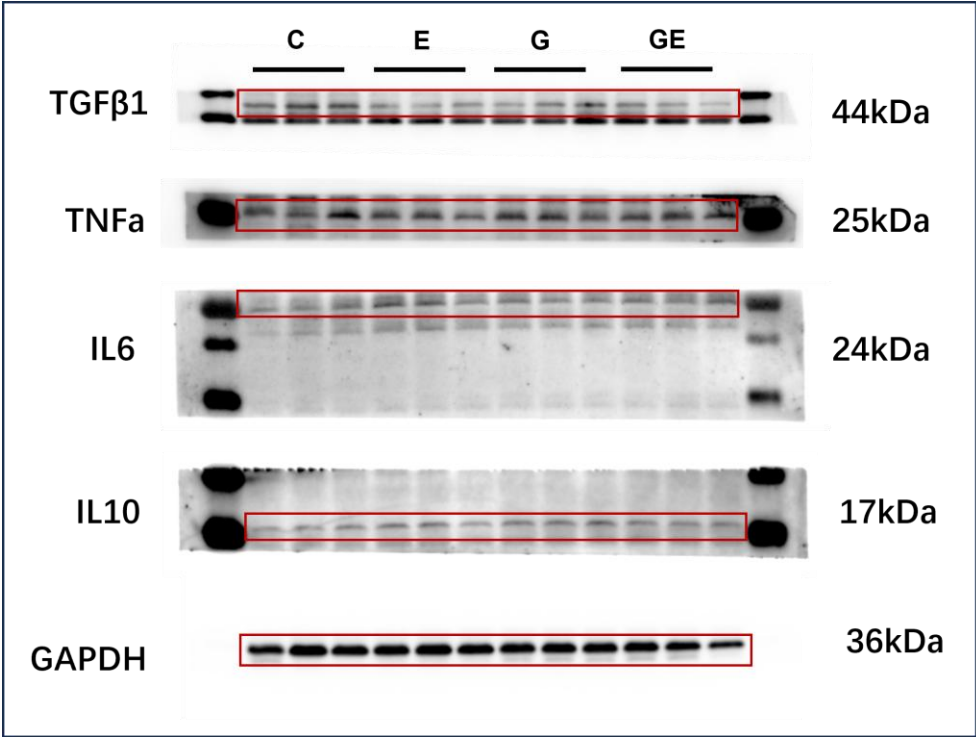

Original image for Fig 3A

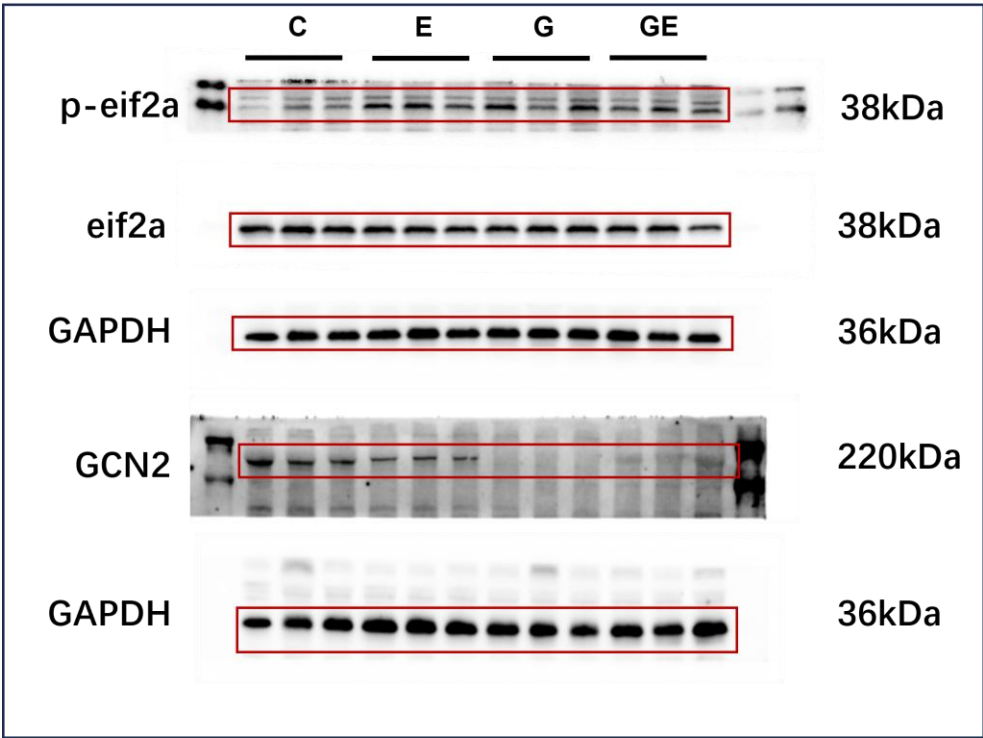

Supplement: S1 Fig — Original images for blot and gel. (PDF) [file pone.0329973.s001.pdf]
